# Perioperative outcome of minimally invasive stabilisation of bilateral fragility fractures of the sacrum: a comparative study of bisegmental transsacral stabilisation versus spinopelvic fixation

**DOI:** 10.1007/s00068-022-02123-6

**Published:** 2022-10-18

**Authors:** Thomas Mendel, Bernhard W. Ullrich, Philipp Schenk, Gunther Olaf Hofmann, Felix Goehre, Stefan Schwan, Florian Brakopp, Friederike Klauke

**Affiliations:** 1 Department of Trauma and Reconstruction Surgery, BG Klinikum Bergmannstrost Halle, Merseburger Strasse 165, 06120 Halle, Germany; 2 Department of Trauma, Hand and Reconstructive Surgery, University Hospital Jena, Jena, Germany; 3 Research Executive Department, BG Klinikum Bergmannstrost Halle, Halle, Germany; 4 Department of Neurosurgery, BG Klinikum Bergmannstrost Halle, Halle, Germany; 5 Department of Biological and Macromolecular Materials, Fraunhofer Institute for Microstructure of Materials and Systems IMWS, Halle, Germany

**Keywords:** Fragility fracture of the pelvis, Bilateral fragility fracture of the sacrum, Geriatric, Bisegmental transsacral stabilization, Spinopelvic fixation, Short-term outcome, Blood loss, Complications

## Abstract

**Purpose:**

Pelvic fragility fractures have steadily risen over the past decades. The primary treatment goal is the fastest possible mobilisation. If conservative therapy fails, surgical fixation is a promising approach. This study compares the outcome of bisegmental transsacral stabilisation (BTS) and spinopelvic fixation (SP) as minimally invasive techniques for bilateral fragility fractures of the sacrum (BFFS).

**Methods:**

We performed a prospective, non-randomised, case-controlled study. Patients were included if they remained bedridden due to pain despite conservative treatment. Group assignment depended on sacral anatomy and fracture type. The outcome was estimated by blood loss calculation, cut-seam time, fluoroscopy time, complications, duration of stay at the intensive/intermediate care unit (ICU/IMC), and total inpatient stay. The mobility level at discharge was recorded.

**Results:**

Seventy-three patients were included (SP: 49, BTS: 24). There was no difference in blood loss (BTS: 461 ± 628 mL, SP: 509 ± 354 mL). BTS showed a significantly lower cut-seam time (72 ± 23 min) than SP (94 ± 27 min). Fluoroscopy time did not differ (BTS: 111 ± 61 s vs. 103 ± 45 s). Thirteen percent of BTS and 16% of SP patients required ICU/IMC stay (BTS: 0.6 ± 1.8 days, SP: 0.5 ± 1.5 days) during inpatient stay (BTS: 9 ± 4 days, SP: 8 ± 3 days). Fourteen patients suffered from urinary tract infections (BTS: 8%; SP: 25%). In-patient mortality was low (BTS: 4.2%, SP: 4.1%). At discharge, the BTS group was almost back to the initial mobility level. In SP patients, mobility was significantly lower than before complaints (*p* = 0.004).

**Conclusion:**

Both methods allow early mobilization of BFFS patients. Blood loss can be kept low. Hence, transfusion requirement is correspondingly low. The IMC/ICU and the total inpatient stay are lower than reported in the literature. Both BTS and SP can be recommended as safe and low-complication methods for use in BFFS patients. BTS is superior to SP with respect to surgery duration and level of mobility at discharge.

**Supplementary Information:**

The online version contains supplementary material available at 10.1007/s00068-022-02123-6.

## Introduction

Fragility fractures of the pelvis (FFP) in the geriatric population are steadily increasing since the past few decades [1, 2]. The cause is likely from low-energy trauma such as a fall from a standing or walking position. In addition, it is not uncommon for elderly patients to suffer such fractures owing to pronounced osteoporosis under physiological repetitive strain without an actual accident event [3]. The primary treatment goal is the fastest possible mobilization. The general consensus is that conservative treatment with demand-oriented pain therapy is the first-line therapeutic approach [4]. If conservative therapy fails, surgical fracture stabilization as a second-line therapeutic step is a promising approach [5].

In cases of FFP, purely transsacral fractures occur in up to two-thirds of cases [6] and thus represent the most common fracture location at the posterior pelvic ring. In addition, there is increasing evidence of a progressive, step-by-step course of the injury, whereby after a primary unilateral sacral fracture, a secondary reciprocal fracture manifests itself within a few weeks [3]. The incidence of such bilateral fragility fractures of the sacrum (BFFS) is 15.1% [6]. An interconnecting transverse fracture component (TFC) often manifests itself in addition to the vertical fracture lines as an indication of increasing instability. Although the spanning ligamentary structures are usually intact, the bilateral discontinuity corresponds to a spinopelvic dissociation.

The literature describes a variety of different surgical stabilisation procedures for the treatment of BFFS. In addition to the classic sacroiliac screw fixation with [7] and without [1] cement augmentation, transsacral stabilization [5, 8] using extra-long screws or sacral bars and sacroplasty [9, 10] is used as minimally invasive procedures. Spinopelvic fixation using open or percutaneous techniques [5] has also been described. The chosen osteosynthesis approach must be able to bear full weight immediately to ensure the fastest possible mobilisation of the geriatric patient. In particular, the duration and extent of surgical intervention and the associated blood loss influence the immediate postoperative course, the rate of any complications, and thus the short-term outcome. Therefore, there is a need for prospective data regarding the extent to which different surgical procedures influence the short-term outcome.

Hence, the aim of this study was to compare the perioperative outcome of the bisegmental transsacral stabilisation (BTS) and spinopelvic fixation (SP) as two specific minimally invasive fixation techniques of the posterior pelvic ring in BFFS with regard to surgical side influences on blood loss, complications, postoperative mobility, and social integrity.

## Patients and methods

In this single-centre, prospective, non-randomised, case-controlled study (evidence level 2), the perioperative outcome results of patients with minimally invasive, stabilised BFFS (FFP4b) [6] were investigated. This included BFFS with or without a TFC in the sense of U- or H-shaped fracture pattern. Two different minimally invasive surgical techniques were compared with regard to surgery-dependent influences on patients’ perioperative outcome. The choice of osteosynthesis depended on the individual sacral anatomy and the fracture types described above [5]. Thus, no case randomization was performed.

**Fig. 1:**
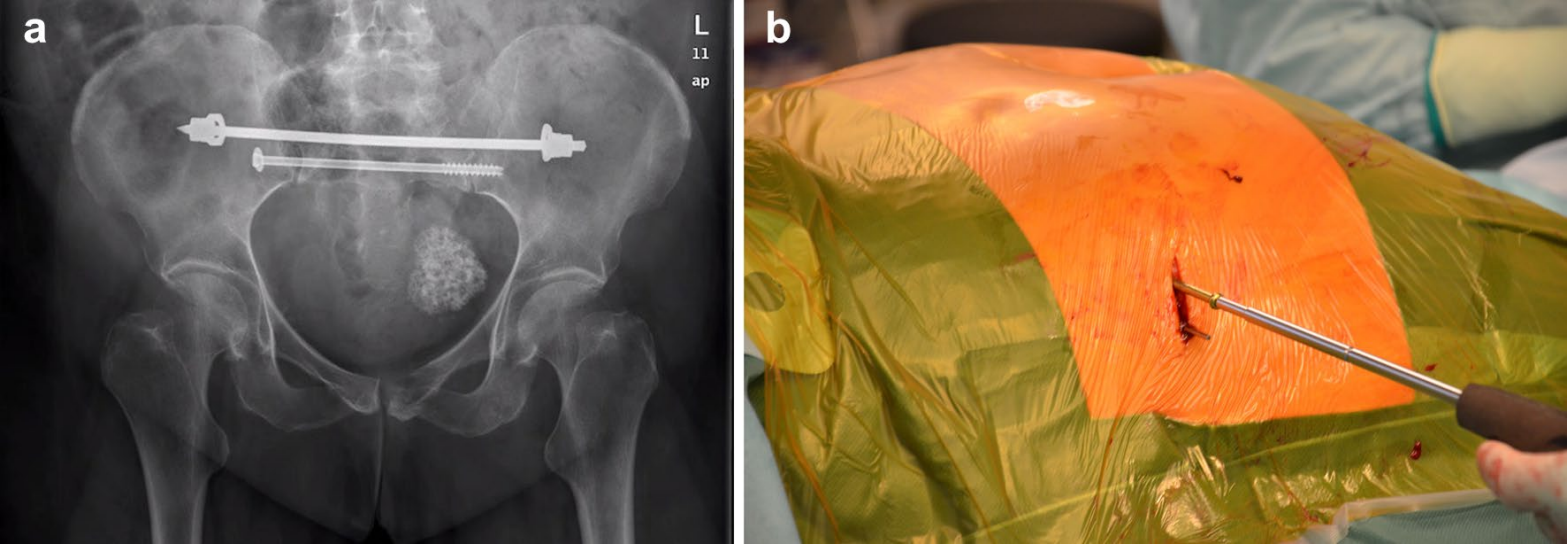
a and b X-ray pelvic overview (**a**) and intraoperative image (**b**) of a bisegmental transsacral screw fixation

Bisegmental transsacral fixation was used whenever two transsacral bone corridors at the S1 and S2 segment level could be used for fracture fixation (Fig. 1a, b). For this purpose, the transsacral bone corridor was visualised by means of a C-arm in a strictly lateral projection of the sacrum. A 2.8-mm wire was then placed on the outer iliac surface through a 2-cm incision and driven in with a hammer so that it projected as a point centrally in the corridor in the lateral fluoroscopic image. Now, the wire was advanced transsacrally step by step under intermittent control in inlet and outlet projection. After overdrilling the wire, the implant was inserted. Either 6.0-mm sacral bars with lock nuts (Depuy-Synthes, Oberdorf, Switzerland) or 7.3-mm cannulated screws (Depuy-Synthes, Zuchwil, Switzerland) were used. With the aim of compressing the alar fracture zones, the ilium was reamed to 6 mm on both sides when sacral bars were used. When 7.3-mm screws were used, a 32-mm partial thread was chosen.

**Fig. 2:**
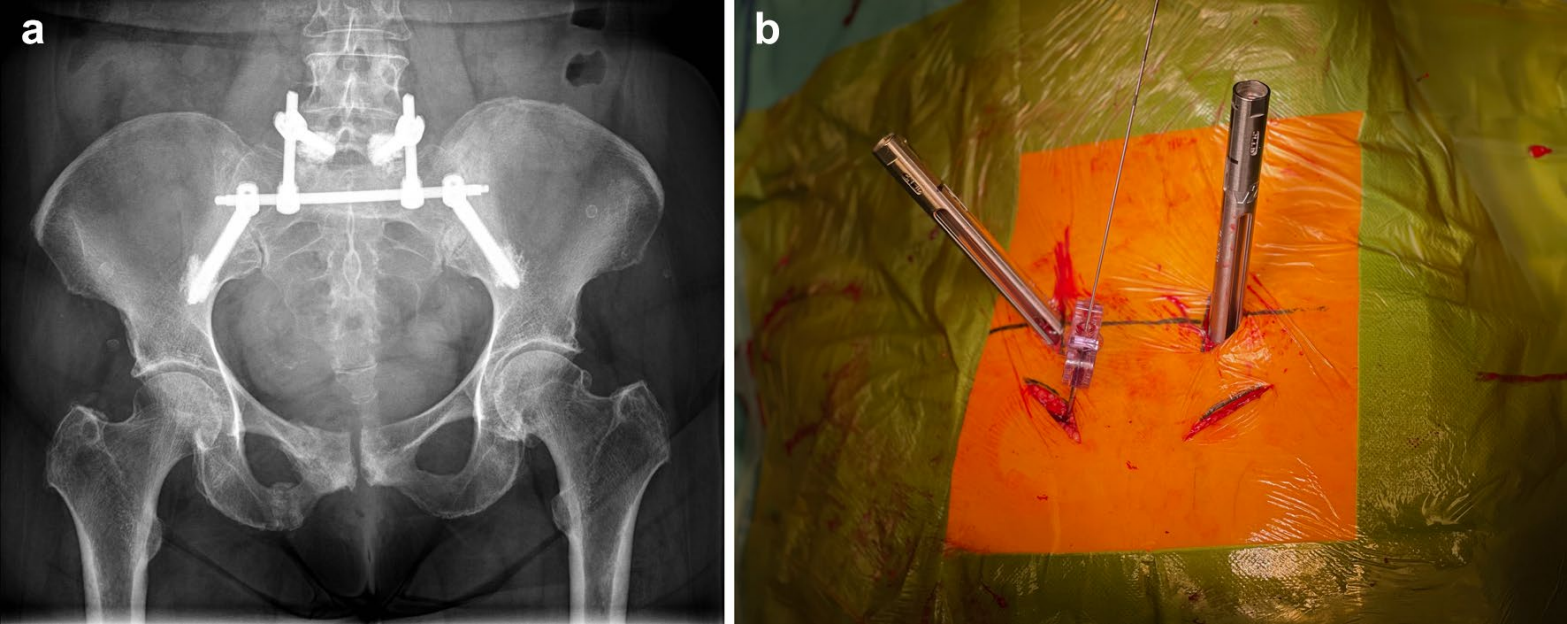
a and b X-ray pelvic overview (**a**) and intraoperative image (**b**) of a spinopelvic fixation

If an H-type fracture with TFC below the first sacral corpus or a sacral dysplasia was present [11, 12] such that either no or only one safe transsacral corridor existed, SP was performed from the fifth lumbar vertebra to both iliac wings (Fig. 2a, b). The internal fixator system Viper 2 (Depuy, Raynham, USA) was used. Polyaxial screws with a diameter corresponding to the pedicle were inserted in L5 using a percutaneous technique via stab incisions. At the ilium, polyaxial screws with a diameter of 9 or 10 mm and a length of at least 80 mm were inserted via 3-cm incisions at the level of the posterior superior iliac spine. The iliac screws were connected horizontally by a 5.5-mm rod inserted subfascially over the guide sleeves. The connection to L5 was then made by inserting lateral connectors (Depuy Expedium 5.5 Spine System, Raynham, USA).

All patients were operated in prone position. Additional stabilisation of the anterior pelvic ring was consistently omitted, as the authors are of the opinion that in FFP, the ligamentary integrity is usually not impaired in a relevant manner due to the low-energy trauma.

Between 2015 and 2018, a total of 124 BFFS patients were treated at our hospital. The diagnosis was usually confirmed with a pelvic radiography and computed tomography. In some cases, a contralateral sacral lesion was detected only after additional magnetic resonance imaging (MRI). Conservative treatment was initially attempted in all cases with the aim of early functional full weight-bearing under adequate pain medication WHO Level 3 for a total of 3–5 days [5]. In case of persistent pain-related immobility, the indication for surgical therapy was determined. After patient inclusion was completed, all patients were allocated to the respective surgical procedure according to the above-mentioned criteria. The study was approved by the independent Medical Ethics Committee of the Medical Council of Saxony-Anhalt, Germany, and confirmed under approval no. 32/16.

### Before start of complaints

Epidemiological data such as age, sex, height, and weight were used to assess group equality. During inpatient admission, the patients were interviewed about their individual life situation and social integrity. The physical condition was assessed with the Modified Frailty Index 5 (mFI5) [13]. In addition, the mobility level, judged according to the need for any orthopaedic walking aids (i.e., orthopaedic aids, crutches, walking bench, high walker, wheelchair, or bedridden state), was recorded using a Likert scale from 0 (worst) to 5 (best) at the time before the start of the complaint.

### Perioperative phase

At the time of study enrolment, all patients were by definition immobile because of pain despite adequate medical analgesia. The individual outcome was estimated by evaluation of surgical-side effects reflected in the perioperative circulation management. The loss of blood volume (BV_loss_) was calculated based on both pre- and postoperative haemoglobin values (Hb_pre_, Hb_post_), body height and weight, and the number of intra- and postoperative transfused red cell concentrates (RCC). Therefore, the following method was applied in three steps. First, individual blood volume (BV) was calculated using the formula of Nadler et al. [14]. Second, the perioperative loss of red cells (RC_loss_) was charged based on the formula of Good et al. [15] assuming that one RCC contains 40 g of haemoglobin. Last, perioperative BV_loss_ was calculated using the quotient of RC_loss_ and the preoperative Hb. Furthermore, perioperative process data like cut seam time (*t*_CS_) and fluoroscopy time (*t*_fluoro_) as well as complications during the surgery were registered for both groups.

### Postoperative inpatient course

Demand and duration of treatment on an intensive/intermediate care unit (ICU/IMC) as well as total duration of inpatient stay were recorded to reflect the impact of surgical intervention to postoperative outcome. Complications occurring during this phase were also recorded. The accommodation status following hospitalisation and the mobility level at the time of discharge was recorded to assess the treatment-related impairment of patient’s independency.

### Statistical analysis

To compare the ratio of sexes between the BTS and SP cohorts, Pearson’s Chi-square test (Fisher exact) was used. For the comparison of epidemiological data (age, body height and weight, BMI); mFi5; mobility level; and Hb_pre_; a multivariate, general linear model (GLM) was used to check differences between both groups. If preoperative differences were found, variables were used to account for bias in the subsequent analysis. By analysing the effect of the surgery on dependent variables (mFi5, mobility level, tCS, tfluoro, Hb_post_, BV_loss_, and duration of ICU/IMC stay), a multivariate GLM was established. Post hoc pairwise comparison was used to check for group differences in dependent variables. Differences between Hb_pre_ and Hb_post_ were compared using separate GLM with repeated measures (rmGLM) with the BTS/SP group variable as the within subject factor. If either method is shown to be superior, a significant interaction effect would be expected. Differences in transfusion frequency, number of RCC, and complications were checked using Pearson’s Chi-square test (Fisher exact). Differences in the mobility level between the time before complains and at discharge were analysed separately for each group with the Wilcoxon signed rank test. Differences of the mobility level between both groups were analysed separately for the time points in accordance with the above Mann–Whitney *U* test. Therefore, the data are given as median values with the interquartile range. The level of significance was set to *p* = 0.05. For all statistical analyses, SPSS (IBM Corporation, Armonk, NY, USA) software was used.

## Results

Out of 124 patients with BFFS, 73 were indicated for surgical therapy given the ongoing pain-related immobility after conservative treatment. According to the above-mentioned criteria for individual fracture morphology and sacral anatomy, 49 and 24 patients, respectively, were treated with SP and BTS. Overall, 89% (*n* = 65) were female. In terms of descriptive data, there was no difference between the two patient groups (Table 1).

**Table 1:** Descriptive data of bisegmental transsacral stabilization (BTS) and spinopelvic (SP) fixation group

	BTS (*n* = 24)	SP (*n* = 49)	*p* value
Male/Female	2/22	6/43	0.713
Age (years)	78 ± 12	80 ± 8	0.427
Height (cm)	162 ± 6	164 ± 7	0.215
Weight (kg)	70 ± 14	73 ± 18	0.491
BMI (kg/m²)	27 ± 5	27 ± 5	0.907
mFI5 (%)	38 ± 18	39 ± 19	0.787

### Before start of complaints

Twenty-two (92%) patients in the BTS group lived independently at home. Two patients (8%) lived in assisted living facilities. In the SP group, 44 (90%) patients lived independently at home, three (6%) were in assisted living, and two (4%) patients had previously lived in a nursing home. No significant differences could be found between both groups. The dependence on orthopaedic aids in the time before the onset of complaints compared to the time of discharge is presented later in the text.

### Perioperative phase

**Fig. 3:**
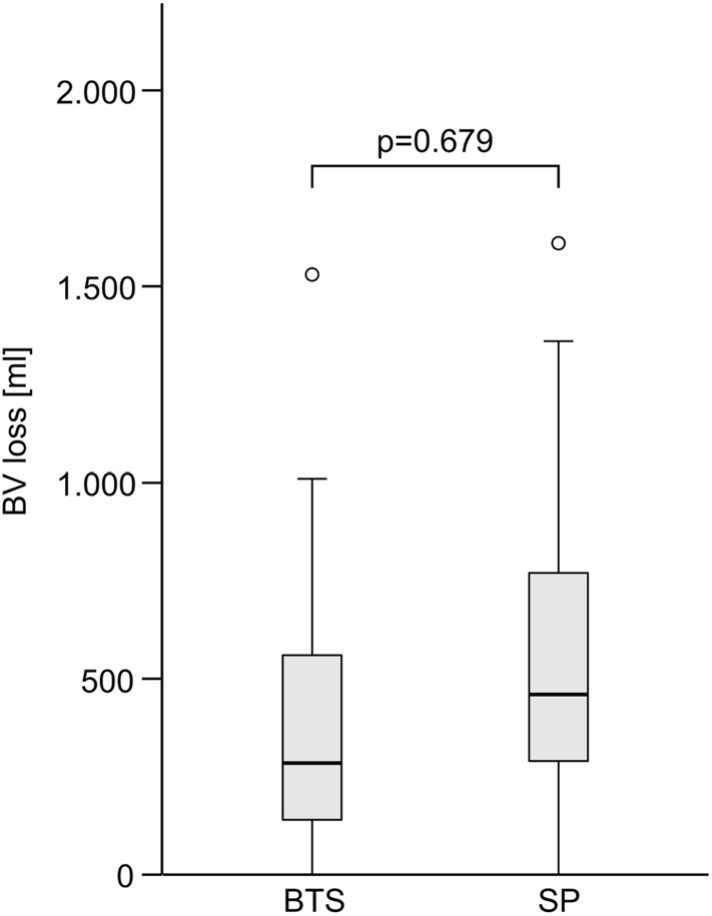
Graph showing comparison of BV_loss_ in the BTS and SP group

All patients were bedridden because of pain at the time of study inclusion. The preoperative laboratory analysis showed comparable Hb_pre_ values in both groups without significant difference (*p* = 0.059, GLM). The rmGLM confirmed significantly lower Hb_post_ in both the BTS and SP groups (*p* < 0.001). However, there was no significant difference between the two surgical methods (*p* = 0.059), and no significant interaction effect was found (*p* = 0.590). The calculated BV_loss_ was low for both techniques (BTS: median 285 mL (IQR [140–560], SP: median 460 mL [280–775]), as expected, and also showed no significant difference in the group comparison (Fig. 3). The need for transfusion was extremely low in both groups. Transfusion was necessary in only two (8%) BTS patients. Two RCCs were administered to each of them. Four (18%) SP patients were transfused, where twice 2, once 1, and once 4 RCCs were given. Both the transfusion frequency and number of RCCs showed no significant difference between the groups (*p* = 0.821).

**Fig. 4:**
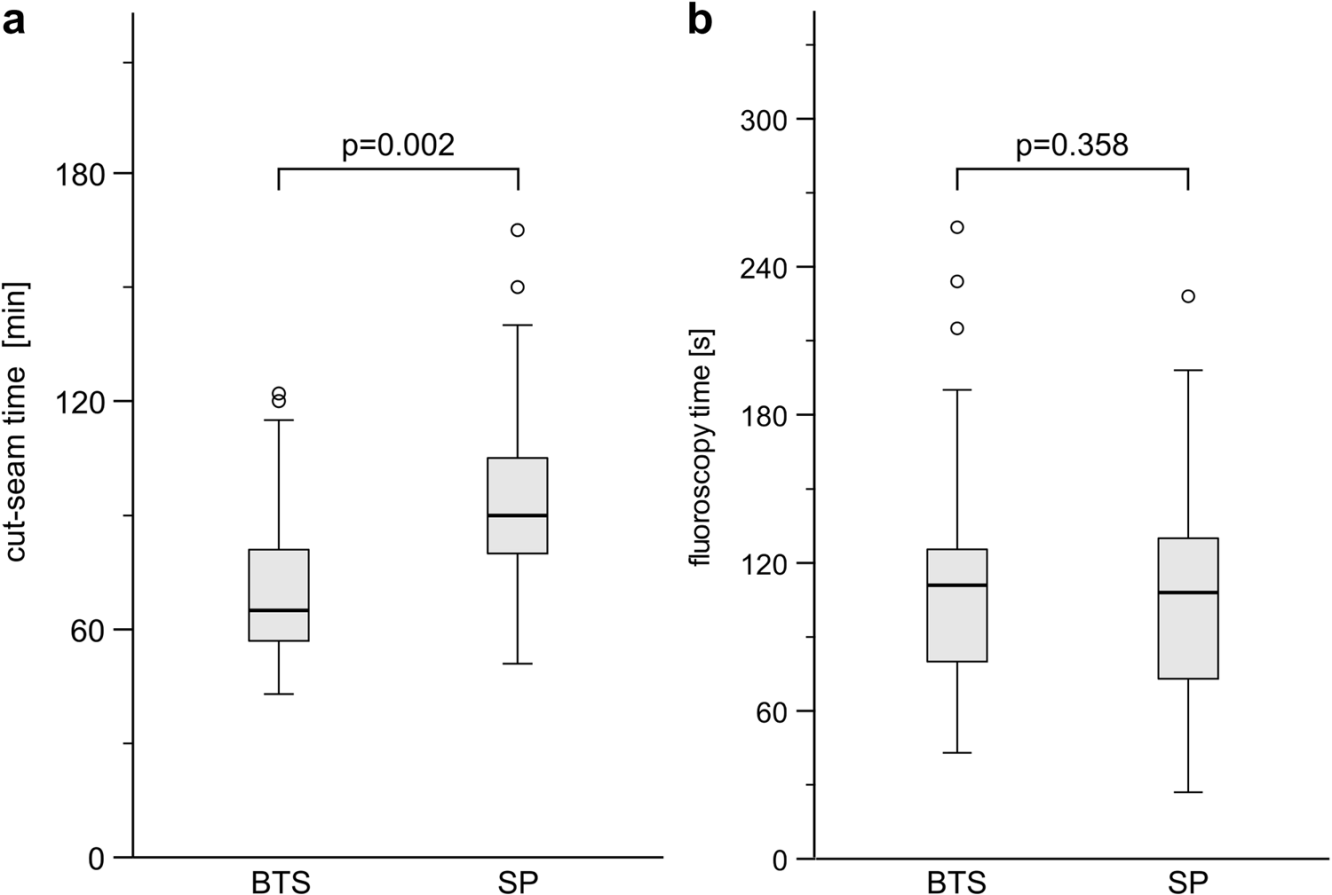
a and b Graph showing significant differences of **a** cut-seam time and **b** fluoroscopy time between both groups

All 73 surgical procedures (SP: 49, BTS: 24) were performed by three experienced pelvic surgeons either by themselves (total: 44, SP: 29, BTS: 15) or as a supervising assistant during training procedures (total: 29, SP: 20, BTS: 9), each of whom has an annual caseload of at least 20 pelvic procedures per year beyond this study. The comparison of the cut-seam time (*t*_cs_) showed a significantly shorter duration in the BTS group (72 ± 23 min) than the SP group (94 ± 27 min) (*p* = 0.002). In contrast, the fluoroscopy time did not differ significantly between the two procedures (111 ± 61 s in the BTS group vs. 103 ± 45 s in the SP group; *p* = 0.358) (Figs. 4a, b).

In the intraoperative course, there were no complications with either of the two surgical methods.

### Postoperative inpatient course

**Fig. 5:**
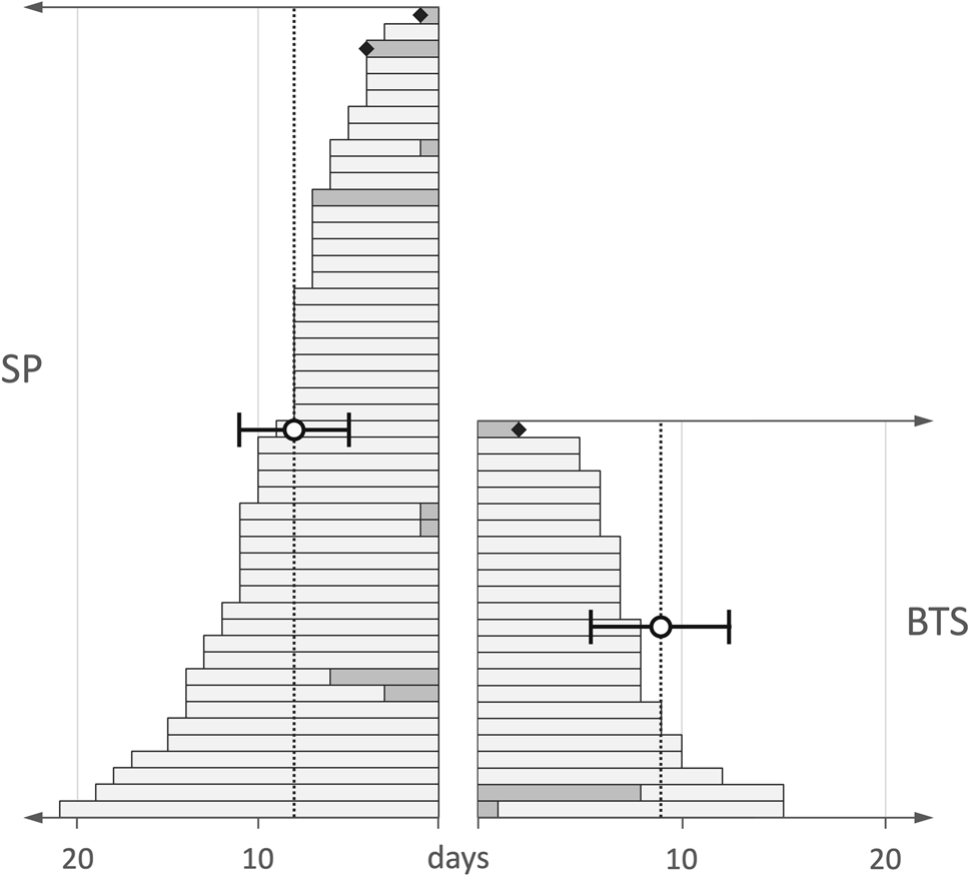
Postoperative inpatient length of stay of both groups with/without the need for treatment in an ICU/IMC ward. Bars represent the postoperative days for the individual patients (dark grey—ICU/IMC, light grey—normal ward). The error bars show the mean and standard deviation within the cohort. The rhombus symbolises a patient’s death

In the total cohort, 62 (85%) patients could be transferred back to the peripheral general ward directly after surgery. Eleven (15%) patients (8/49 [16%] in the SP group and 3/24 [13%] in the BTS group) required temporary care in an ICU/IMC postoperatively, but no significant differences were noted (*p* = 1.000). The average ICU/IMC stay was 0.5 ± 1.5 days in the SP group and 0.6 ± 1.8 days in the BTS group (*p* = 0.851). The mean postoperative inpatient length of stay did not differ significantly between SP and BTS (8 ± 3 days vs. 9 ± 4 days, [*p* = 0.143]) (Fig. 5).

Neither in the SP nor in the BTS group implant malposition or neurological complications occurred. Preoperatively, a total of 12 patients were on anticoagulant medication with coumarin derivatives or direct oral anticoagulants due to previous cardiac diseases (SP: 11, BTS: 1). Of these, six patients could be switched preoperatively to a low molecular weight heparin in a prophylactic dose. In six other cases, surgery had to be performed under therapeutic anticoagulation (SP: 6, BTS: 0). One of these developed a wound haematoma requiring revision surgery. Implant-associated complications or infections did not occur in either group. Fourteen patients suffered from uncomplicated urinary tract infections (BTS: *n* = 2 [8%]; SP: *n* = 12 [25%], *p* = 0.124). No patient experienced postoperative prerenal renal failure. One BTS patient died of an acute pulmonary embolism during the inpatient stay. In the SP group, one patient died because of an acute gastric perforation, having previously knowingly refused emergency surgery. Another SP patient died of an acute thromboembolic event. Thus, perioperative in-patient mortality was relatively low (BTS: 4.2%, SP: 4.1%).

**Fig. 6:**
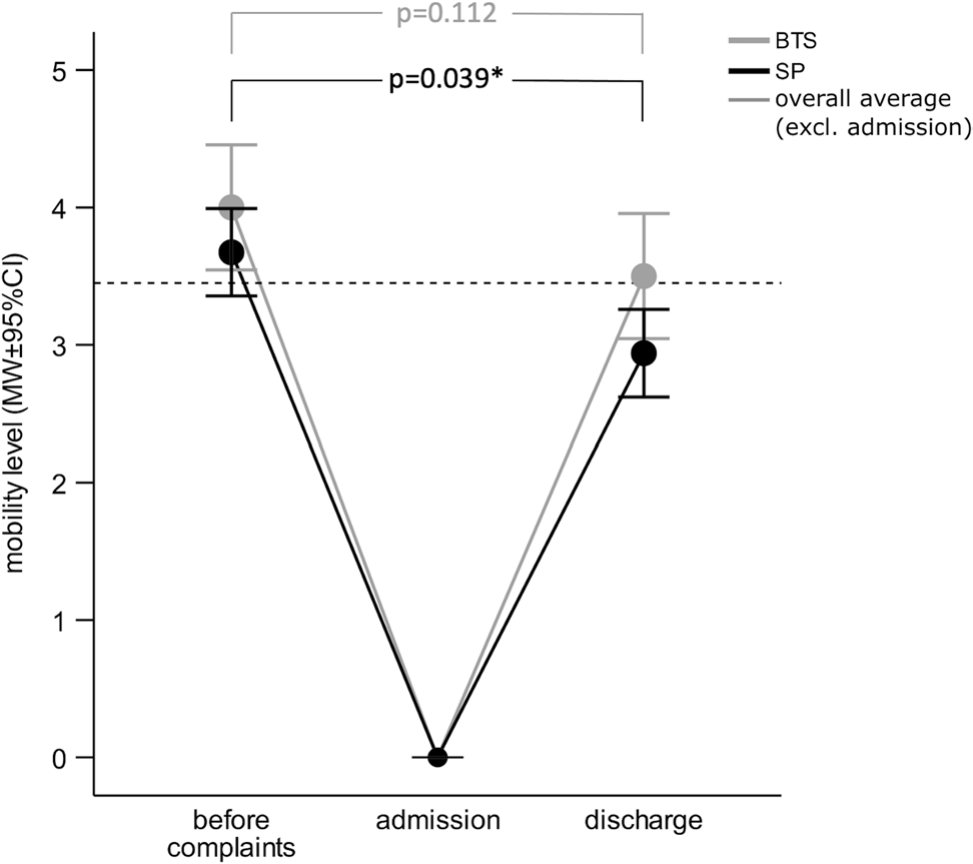
Comparison of mobility level of SP and BTS groups related to examination time points

Before the onset of complaints (TP1), there was no significant difference between the groups in terms of mobility (BTS: 4.0 [3.3–5.0] versus SP: 4.0 [2.0–5.0], *p* = 0.422). At the time of hospital admission (TP2), all patients were bedridden by definition. By the day of discharge (TP3), the majority could be mobilized in terms of standing and gait. However, at TP3, the median of the BTS group was almost back to the initial level of mobility (TP1) with no statistical difference between TP1 and TP3 (4.0 [3.3–5.0] versus 3.5 [3.0–4.0], *p* = 0.112), which is why the mobility achieved in this respect was higher than with SP (*p* = 0.039). Although patients in the SP group were already mostly mobile again by TP3, the mobility level was significantly lower than at TP1 (4.0 [2.0–5.0] versus 3.0 [2.0–4.0], *p* = 0.004) (Fig. 6). In summary, only one BTS patient (4.3%) and six SP patients (12.5%) were dependent on a wheelchair. No patient (0%) was bedridden.

At TP1, 97% of the entire population lived at home. At TP3, 19% of all patients could be discharged directly to the home environment. 50% of all patients could be transferred to an inpatient rehabilitation facility with good rehabilitation potential. Due to comorbidities requiring treatment or an increased need for care, 25% were transferred to an acute geriatric ward or short-term care. Table 2 gives an overview of the group-related distribution.

**Table 2:** Overview of the living conditions before the onset of complaints and after the inpatient treatment in relation to and the surgical method used

Before complaints		Living conditions	At discharge	
SP	BTS		SP	BTS
44 (90%)	22 (92%)	Independent at home	5 (10%)	8 (33%)
3 (6%)	2 (8%)	Assisted living	1 (2%)	
2 (4%)		Nursing home	1 (2%)	2 (8%)
		Inpatient rehab institution	26 (53%)	9 (38%)
		Acute geriatric care	11 (22%)	
		Short time care	3 (6%)	4 (17%)
		Death	2 (4%)	1 (4%)

## Discussion

The perioperative outcome of geriatric patients with FFP is primarily limited by the underlying individual comorbidities. This can only be positively influenced to a limited extent during acute inpatient treatment. Therefore, surgical treatment should aim at mobilizing patients as quickly as possible to prevent secondary complications such as pneumonia, thrombosis, and urinary tract infections. This includes adequate fracture stabilisation for sufficient relief of symptoms. At the same time, surgical invasiveness and perioperative blood loss should be kept to a minimum to prevent haemorrhagic circulatory depression with resulting organ dysfunction such as prerenal renal failure [16].

Regarding minimally invasive stabilization of pelvic fractures, Riesner et al. described an external blood loss of only 70 mL on average in a study of 23 triangular stabilizations [17]. Blake-Toker et al. even quoted a mean blood loss of < 10 mL for CT-navigated SI screw fixation [18]. Similar results were found by Long et al. for SI screws with 43 mL without and 33 mL with TiRobot support [19]. In summary, blood loss for minimally invasive posterior pelvic ring stabilisation techniques appears to be negligible. However, external blood loss in the context of surgical interventions is routinely measured by the volume drained in the aspirator and collected in gaze dressings, but this does not take into account the volume of blood lost intracorporeally in the interstitial tissue [15, 19]. This is the advantage of calculation of perioperative blood loss based on the pre- and postoperative Hb balance capturing both the external and internal blood loss that occurs in the time window between the pre- and postoperative blood collection [20]. For example, Kohler et al. used this method in patients with unstable thoracolumbar vertebral fractures who underwent minimally invasive multilevel dorsal stabilisation, and found a mean blood loss of 700 mL [16]. Good et al. calculated a mean blood loss of 1426 mL for the implantation of knee endoprostheses [15]. In an Australian benchmark study with 1,296 patients, a blood loss of 35.2% of the preoperative total blood volume was determined [20]. In our study, the external and internal blood loss was determined for the first time in relation to the surgical treatment of BFFS using the Hb balance. A mean blood loss of approximately 500 mL could be calculated for each of the two surgical methods compared. The resulting benefit in the postoperative outcome for both methods is reflected in a comparably low need for transfusions and either did not or only briefly required postoperative intensive medical care and a short overall postoperative stay. There is no comparable data in the literature for FFP for both surgery-related transfusion requirement and the need for intensive care.

The duration of surgery is considered a trigger for the incidence of secondary complications, especially in elderly patients. Consequently, surgery should be kept as short as possible. Vanderschot et al. reported a mean operation time of 60 min for the implantation of a single transsacral rod in 19 patients [16]. In our cohort of 24 geriatric patients, the insertion of two transsacral bars took an average of only 72 min. For spinopelvic fixation, we required 111 min. In contrast, Koshimune et al. required a mean of 208 min for a minimally invasive SP of traumatic pelvic ring injuries [21]. The minimally invasive surgical procedure aims to reduce the iatrogenic soft tissue trauma. However, in the posterior pelvic ring, especially, an exclusive and even more limited fluoroscopic orientation places high demands on the surgeon’s skills. For the classic sacroiliac (SI) screw fixation, an average fluoroscopy time of 1.8–2.3 min per SI screw inserted has been reported in the literature [22, 23]. The BTS we used functionally corresponds to the insertion of a total of four SI screws, whereby a mean total fluoroscopy time per operation of only 1.85 min was required. A comparable level of radiation exposure of only 1.71 min was also achieved with SP. Consequently, we were able to show that both standardised methods are basically associated with a short duration of surgery and low radiation exposure.

The length of inpatient stay correlates significantly with the loss of independence, the degree of care dependency, and the rate of institutionalized accommodation [24]. Hopf et al. reported about 30 patients with FFP treated with SI screw fixation [1]. The average hospital stay was 23.7 days. Noser et al. described a mean hospitalization period of 13 days (range 2–78 days) in surgical treated fragility fractures of the sacrum [25]. Vanderschot et al. reported a length of stay of only 5 days for transsacral stabilisation [26]. Kanakaris et al. described a mean length of stay of 22.4 days, but included both conservatively and surgically treated FFP patients [27]. Gericke et al. described a postoperative inpatient stay of 9.7 days for minimally invasive stabilised FFP [28]. However, all FFP fracture types were included in a total of 379 cases. Only 14.5% were BFFS of type FFP4b. Rommens et al. summarised the results of FFP2, 3, and 4 lesions and reported a mean length of stay of 8 days in conservatively treated patients. In contrast, they reported a length of stay of 18 days after surgical treatment [29]. Although in our study only patients with highly unstable FFP4b were included, a comparably short length of stay of 8 (SP) and 9 days (BTS) was achieved with both surgical techniques.

Intraoperative and secondary complications can significantly affect the perioperative outcome. Gericke et al. recorded a general complication rate of 28.4% in 74 minimally invasive treated FFP patients, primarily urinary tract infections. The reported rate of surgical-side complications was 9.5%, and the leading cause was implant failure in 6.8% of cases [28]. Rommens et al. also reported a high rate of urinary tract infections (36.1%). They also reported a 23.1% rate of surgery-induced complications [29]. In our study, urinary tract infections occurred in 8% (BTS) and 25% (SP) patients, showing similar results to the previously mentioned studies. However, with the exception of one haematoma requiring revision after SP, we recorded no surgical-side complications in our cohorts.

The 1-year mortality rate of geriatric patients with FFP is reported in the literature to be between 10 and 27% [5, 29–31] and is therefore comparable to the outcome of hip fractures. Thus, in many cases, FFP is considered an expression of the approaching end of life. Hence, there is consensus that the primary goal of treatment should be to mobilize patients as quickly as possible [5]. Yoshida et al. compared 324 conservatively treated patients with 16 surgically treated FFP patients and described that only 40% of the conservative group were mobile in stance and gait after 1 year. In contrast, 78% of 16 operated patients were mobile after 1 year [32]. Rommens et al. studied the perioperative outcome of 138 patients treated surgically. They summarised FFP types 2, 3, and 4 and reported that 56.1% were able to walk on an inpatient level at the time of discharge [29]. Our study shows that even in FFP4b fracture types, minimally invasive fixation allows rapid mobilisation under immediate pain-oriented full weight-bearing just within an inpatient treatment phase of 8 days (SP) and 9 days (BTS), where 96% and 88% of the patients, respectively, were mobile in stance and gait using at least a walking bench or a rollator. At the time of discharge, BTS patients even reached the mobility level that was indicated before the onset of complaints, which indicates an advantage over SP.

Only a few studies describe the course of treatment immediately following the acute inpatient stay. Kanakaris et al. differentiated FFP patients into the following four categories: unaided community ambulatory, aided community ambulatory, household ambulatory, and non-functional ambulatory. In 56.6% cases, patients were able to regain their pre-injury status after one year, while 32% lost one and 11.3% lost two status levels [27]. Vanderschot et al. analysed 19 patients with sacral insufficiency fractures who were treated using trans-sacral screw fixation. Of these, 16 could be directly discharged home or were transferred to a nursing home. Three patients were transferred to another department for treatment of comorbidities [26]. In another study with 138 surgically treated patients with FFP2, 3, and 4 fracture patterns, 27.5% could be directly discharged home. In 63% cases, patients were transferred to a rehabilitation facility [29]. In our study, 97% patients were still living independently at home before the onset of symptoms. After minimally invasive stabilisation, 19% could be directly discharged home again post-inpatient. Another 50% were transferred to an inpatient rehabilitation institution. Thus, more than two thirds of our patients had a high potential for regaining social independence. Taking into account the mobility achieved postoperatively as well as the individual degree of independence gained, the results in our patient population with FFP4b lesions can be described as very good compared to the literature.

The prospective design of our comparative cohort study is a major strength. In addition, the groups compared are very homogeneous both in terms of epidemiological data and regarding the consideration of one specific fracture type. However, our study also has some limitations. The choice of osteosynthesis procedure was based on the individual anatomy of the sacrum and the described fracture types [5]. Thus, no case randomisation was performed. Furthermore, our presented case numbers are small and therefore only allow a limited interpretation of the results. In addition, anticoagulant therapy influences the extent of perioperative blood loss, which must be taken into account when interpreting the results of the Hb balance. Thus, one out of six patients who underwent surgery under therapeutic anticoagulation experienced a bleeding complication requiring revision. On the other hand, there was no significant difference in the extent of blood loss compared to patients with prophylactic anticoagulation using heparin.

## Conclusion

According to the results of this study, an early mobilisation of BFFS patients in stance and gait can already be achieved in the immediate postoperative inpatient setting with both the BTS and SP methods. This is an expression of the fact that both methods can achieve sufficient mechanical stabilisation of the posterior pelvic ring. Due to the minimally invasive procedure in each case, blood loss can be kept low, whereby the calculation based on the Hb balance is a reliable method that takes both external and internal blood loss into account. The transfusion requirement is correspondingly low. The required duration of an intensive care stay as well as a total inpatient stay are low when compared to the reported times in the published literature. Both BTS and SP are recommended to be safe and low-complication surgical methods for use in geriatric patients with FFP4b injuries. However, BTS is superior to SP with respect to the duration of surgery and the mobility level attained at discharge.

## Supplementary Information

Below is the link to the electronic supplementary material.

Supplementary file1 (DOCX 17 KB)
