# Supplementary material for: Perioperative outcome of minimally invasive stabilisation of bilateral fragility fractures of the sacrum: a comparative study of bisegmental transsacral stabilisation versus spinopelvic fixation
Source: Eur J Trauma Emerg Surg. 2022 Oct 18;49(2):1001–10. doi: 10.1007/s00068-022-02123-6 (PMC10175409; doi:10.1007/s00068-022-02123-6)
Supplement: Supplementary file 1 — Supplementary file1 (DOCX 17 KB) [file 68_2022_2123_MOESM1_ESM.docx]

**Supporting information**

**Figures 1a and b**

X-ray pelvic overview (a) and intraoperative image (b) of a bisegmental transsacral screw fixation

**Figures 2a and b**

X-ray pelvic overview (a) and intraoperative image (b) of a spinopelvic fixation

**Figure 3**

Graph showing comparison of BV_loss_ in the BTS and SP group

**Figures 4a and b**

Graph showing significant differences of (a) cut-seam time and (b) fluoroscopy time between both groups

**Figure 5**

Postoperative inpatient length of stay of both groups with/without the need for treatment in an ICU/IMC ward. Bars represent the postoperative days for the individual patients (dark grey - ICU/IMC, light grey - normal ward). The error bars show the mean and standard deviation within the cohort. The rhombus symbolises a patient’s death.

**Figure 6**

Comparison of mobility level of SP and BTS groups related to examination time points.

**Table 1**

Descriptive data of bisegmental transsacral stabilization (BTS) and spinopelvic (SP) fixation group

**Table 2**

Overview of the living conditions before the onset of complaints and after the inpatient treatment in relation to and the surgical method used
